# GH Stimulation testing is unnecessary in patients with hypothalamic–pituitary disease, preserved pituitary function, and IGF-I SDS ≥ 0

**DOI:** 10.1007/s11102-026-01697-3

**Published:** 2026-07-23

**Authors:** Valentina Gasco, Daniela Cuboni, Michela Sibilla, Francesca Mocellini, Alice Martinotti, Davide Lucisano, Ezio Ghigo, Gianluca Aimaretti, Silvia Grottoli, Mauro Maccario

**Affiliations:** 1 Division of Endocrinology, Diabetes and Metabolism, Department of Medical Sciences, University of Turin, Turin, Italy; 2 Division of Endocrinology, Diabetes and Metabolism, Department of Medical Sciences, “Città della Salute e della Scienza” Hospital, Corso Dogliotti 14, Turin, 10126 Italy

**Keywords:** Adult growth hormone deficiency, IGF-I, Bayesian analysis, Diagnostic probability, GH stimulation testing, Pituitary function

## Abstract

**Purpose:**

In adults with hypothalamic–pituitary disease, the presence of at least three additional pituitary hormone deficiencies, with or without markedly reduced IGF-I levels, is considered diagnostic of growth hormone deficiency (GHD) without the need for stimulation testing. In all other cases, dynamic testing is required despite its well-recognized limitations. To date, no formal strategy has been proposed to exclude GHD without performing stimulation tests.

This study aims to develop a probabilistic model to estimate the likelihood of GHD in adults with hypothalamic–pituitary disease but no additional pituitary hormone deficiencies, based on IGF-I standard deviation scores (SDS).

**Methods:**

We combined literature-derived data on: (1) the probability of an impaired GH response to stimulation tests in patients with hypothalamic–pituitary disease and preserved anterior pituitary function; and (2) the probability that adults with isolated GHD present IGF-I SDS values ≥ 0, ≥ 0.5, or ≥ 1. A Bayesian approach was applied to estimate the post-test probability of GHD in patients meeting these criteria.

**Result:**

Across all models, the estimated probability of adult-onset GHD ranged from 6.5% to 19.8%, while the probability of childhood-onset GHD ranged from 2.9% to 9.1%, consistently remaining below 20%, regardless of the IGF-I SDS cut-off applied.

**Conclusion:**

In adults with hypothalamic–pituitary disease but no additional hormonal deficiencies, IGF-I SDS values ≥ 0 identify a subgroup with a low, clinically acceptable probability of GHD, in whom stimulation testing may reasonably be avoided. This proof-of-concept model supports a simplified, probability-based diagnostic approach.

## Introduction

Adult growth hormone deficiency (GHD) represents a complex, heterogeneous, and often underdiagnosed clinical condition [1–3]. In adulthood, GHD most commonly results from acquired hypothalamic–pituitary disorders, including pituitary adenomas, craniopharyngiomas, surgical or radiotherapy sequelae, traumatic brain injury, and infiltrative diseases. In addition, a subset of cases reflects persistent childhood-onset GHD, reconfirmed after completion of growth and reassessment in adult life [1, 4–7].

However, the diagnosis of adult GHD may be challenging, as clinical presentation is often subtle and characterized by nonspecific features, including fatigue, impaired quality of life, increased visceral adiposity, reduced lean body mass, dyslipidemia, decreased bone mineral density, and a suspected increase in mortality [1, 5, 7–12].

The diagnosis of adult GHD traditionally relies on dynamic GH stimulation tests, as basal GH concentrations have limited diagnostic value due to their pulsatile secretion and marked circadian variability [2–7, 13, 14]. This approach results in a significant diagnostic burden for both patients and healthcare systems, with increased direct and indirect costs. Among available tests, the insulin tolerance test (ITT) is considered the gold standard, while alternative tests—including the GHRH–arginine test, the glucagon stimulation test, and more recently macimorelin—are used when ITT is contraindicated or not feasible [4–7, 14]. Nevertheless, the routine use of these tests is associated with relevant limitations [2]. The ITT requires intensive monitoring and carries a higher risk profile; the GHRH–arginine test is no longer available following the discontinuation of GHRH production; and alternative tests show variable sensitivity and specificity and lack well-established normative thresholds in overweight and obese individuals, a condition itself associated with blunted GH responses to stimulation [2, 3, 14, 15]. In this context, the complexity of test interpretation may delay diagnosis or lead to overdiagnosis, with important clinical and economic implications.

Serum insulin-like growth factor I (IGF-I), a peripheral and relatively stable surrogate of GH activity, may provide adjunctive diagnostic information in selected clinical contexts [16]. Although normal IGF-I concentrations do not exclude adult GHD, markedly reduced levels (i.e., < −2 standard deviation score [SDS]) are highly suggestive of GHD when interpreted within an appropriate clinical framework [4, 7, 17]. It should be noted, however, that serum IGF-I concentrations exhibit substantial overlap between healthy older individuals and age-matched patients with GHD or hypopituitarism [18–21]. Consequently, while the diagnostic sensitivity of IGF-I is relatively high in adolescents and young adults, it declines significantly beyond 40 years of age [18–21]. Nonetheless, the majority of adults with GHD demonstrate IGF-I concentrations in the lower half of the reference interval (IGF-I SDS < 0) or below the normal range [18, 21]. Therefore, in untreated adults, an IGF-I SDS ≥ 0 renders the diagnosis of GHD unlikely [21].

In addition, long-standing impairment of residual pituitary function has been recognized as a reliable marker of GHD. Several studies have reported that the probability of GHD in adults presenting with at least three additional pituitary hormone deficiencies ranges from approximately 90.7% to 96% [17, 22, 23]. Accordingly, international guidelines state that the presence of three or more pituitary hormone deficiencies, with or without IGF-I SDS levels < − 2.0, may be considered diagnostic of GHD without the need for dynamic testing [4–7].

In recent years, increasing interest has focused on alternative diagnostic strategies aimed at reducing reliance on GH stimulation tests, particularly in clinical contexts where such tests are poorly tolerated or difficult to access. Attention has shifted toward the integration of clinical, biochemical, and anamnestic parameters through predictive models, decision algorithms, or Bayesian-based approaches [21, 24, 25]. The goal of these strategies is to refine pre-test probability estimation, thereby limiting dynamic testing to cases in which it is most likely to provide meaningful diagnostic information. Despite these conceptual advances, validated and widely accepted models to support diagnostic decision-making in routine clinical practice are still lacking.

Within this framework, the evaluation of IGF-I levels and their integration with pre-test probability estimates represents a concrete opportunity to improve diagnostic efficiency. Identifying subgroups of patients in whom GHD can be reasonably excluded without pharmacological stimulation would offer substantial benefits for both patients and healthcare systems, while allowing diagnostic efforts to be focused on more complex cases.

The present study fits within this evolving diagnostic paradigm and aims to estimate the probability of GHD in adults with hypothalamic–pituitary disease but no additional pituitary hormone deficiencies, based on IGF-I SDS levels, using a Bayesian mathematical model. The ultimate goal is to propose an approach that integrates existing evidence and contributes to the development of clearer and more rational criteria for the diagnostic management of these patients, reducing dependence on dynamic testing and promoting a more efficient use of clinical resources.

## Materials and methods

We analyzed and integrated data available in the literature regarding: (i) the probability of isolated GHD (IGHD)—defined by an impaired GH response to stimulation tests—in adult patients with hypothalamic–pituitary disorders who did not present additional pituitary hormone deficiencies; and (ii) the probability that adult patients with IGHD—defined by an impaired GH response to stimulation tests—presented IGF-I SDS values ≥ 0, ≥ 0.5, or ≥ 1.

### Estimation of the probability of IGHD at GH stimulation testing in the absence of additional pituitary hormone deficiencies

**Fig. 1:**
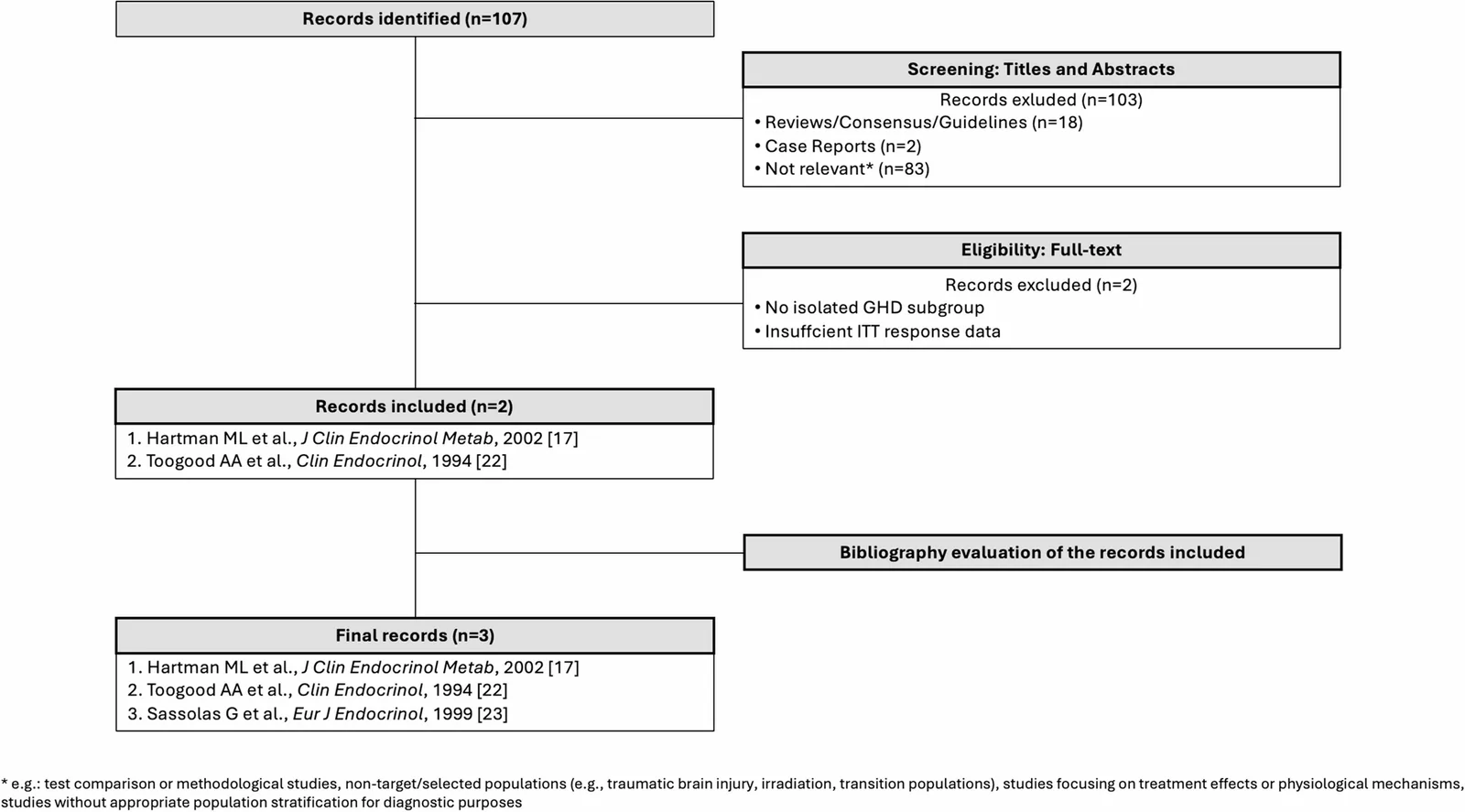
Flowchart of study selection. Studies were identified through database searches and screened based on predefined inclusion and exclusion criteria. Only studies providing data on GH stimulation test outcomes in function of the number of pituitary hormone deficiencies in adults with hypothalamic–pituitary disease were included in the final analysis

A systematic literature search was performed to identify studies reporting the probability of IGHD in adult patients without additional pituitary hormone deficiencies. Only studies conducted in adult subjects, using the ITT as the diagnostic tool and published in English, were included. A PubMed search using the following string: ((pituitary OR hypothalamic OR “hypothalamic-pituitary”) AND (“growth hormone deficiency” OR “isolated GH deficiency”) AND (“insulin tolerance test” OR ITT OR “GH stimulation test”) AND (response OR peak OR cut-off OR abnormal)) retrieved 107 articles. Titles and abstracts were independently screened by two authors (V.G. and D.C.), and reference lists of potentially relevant studies were also examined. Ultimately, three studies met the inclusion criteria and were included in the analysis [17, 22, 23]. Figure 1 illustrates the study selection flowchart.

### Estimation of IGF-I SDS percentiles

To estimate the distribution of IGF-I SDS values in adult patients with IGHD, we relied on data from the KIMS database, the largest international registry of adult GHD patients, which provides standardized and centrally validated biochemical data. Published analyses from this database report mean ± standard deviation (SD) IGF-I SDS values in large cohorts of adult patients with IGHD, stratified according to childhood-onset GHD (CO-GHD) [26] and adult-onset GHD (AO-GHD) [26, 27].

The large sample size and standardized assessment of IGF-I SDS in KIMS ensure stable estimates of distribution parameters, allowing derivation of the corresponding percentile probabilities for the present Bayesian model.

Specifically, based on the mean ± SD IGF-I SDS values reported in patients with IGHD in the KIMS database, the percentiles corresponding to IGF-I SDS values equal to 0, 0.5, and 1 were calculated. It should be noted that IGF-I SDS values are standardized with respect to a healthy reference population and therefore follow a normal distribution by definition only in that context. In the present analysis, however, we considered the distribution of IGF-I SDS within populations of patients with IGHD, in whom the distribution is shifted and may differ in dispersion. Accordingly, the percentiles corresponding to predefined IGF-I SDS thresholds (0, 0.5, and 1) were estimated relative to the IGHD population, based on the reported mean and SD values. This approach does not imply re-standardization of IGF-I SDS, but rather estimation of cumulative probabilities within a disease-specific distribution. This approximation assumes that IGF-I SDS values are approximately normally distributed within the IGHD population; this assumption was considered reasonable given the large sample sizes and the absence of evidence of marked skewness in the reported datasets. This allowed estimation of the probability that a patient with IGHD would present IGF-I SDS values equal to or greater than each of the selected cut-offs.

Based on the mean and SD of IGF-I SDS values observed in the KIMS study population, each predefined IGF-I SDS threshold (0, 0.5, and 1) was expressed in terms of its distance from the population mean in SD units according to the following formula

$$\:\frac{X-\mu\:}{\sigma\:}$$

where X represents the IGF-I SDS value of interest, µ the population mean, and σ the SD.

The corresponding percentile was then derived using the cumulative distribution function of the normal distribution, allowing estimation of the proportion of individuals with values equal to or greater than the threshold considered.

Specifically, the percentiles corresponding to IGF-I SDS values of 0, 0.5, and 1 were calculated by applying the procedure described above and deriving the corresponding cumulative probabilities, expressed as percentiles.

### Estimation of the probability that a patient is truly affected by IGHD in the absence of additional pituitary hormone deficiencies and in the presence of IGF-I SDS ≥ 0, 0.5, or 1

The post-test probability of IGHD (X) in the presence of IGF-I SDS values greater than or equal to a given threshold (Y) was estimated by applying Bayes’ theorem. In this framework, disease X represents the event of interest, whereas condition Y constitutes the observed evidence.

The prior probability of disease X, denoted as $\:P\left(X\right)$, was set to predefined values based on previous studies that estimated prevalence in the reference population [17, 22, 23]. The complementary probability of not having the disease was calculated as $\:P(\neg\:X)=1-P\left(X\right).$

The conditional probability of observing condition Y among subjects affected by disease X, $\:P\left(Y\right|X),$ was treated as a variable and analyzed across different scenarios (IGF-I SDS ≥ 0, ≥ 0.5, or ≥ 1), in order to assess the impact of different sensitivity levels of condition Y with respect to disease X. In parallel, the probability of observing condition Y among subjects not affected by disease X, defined as $\:P\left(Y\right|\neg\:X)$, was taken into account and represented the prevalence of condition Y in the non-affected population. This probability was set at 50% for IGF-I SDS ≥ 0, 31% for IGF-I SDS ≥ 0.5, and 16% for IGF-I SDS ≥ 1.

The marginal probability of observing condition Y in the overall population, $\:P\left(Y\right)$, was calculated using the law of total probability: $\:P\left(Y\right)=P\left(Y\right|X\left)\:P\right(X)+P(Y|\neg\:X)\:P(\neg\:X)$.

Subsequently, the post-test probability of disease X in the presence of condition Y, $\:P\left(X\right|Y)$, was calculated by applying Bayes’ theorem in its canonical form: $\:P\left(X\right|Y)=P(Y\left|X\right)\:P\left(X\right)/P\left(Y\right)$.

By substituting the expression for the marginal probability $$\:P\left(Y\right)$$, the formula becomes: $$\:P\left(X\right|Y)=P(Y\left|X\right)\:P\left(X\right)/$$$$\left[P\right(Y\left|X\right)\:P\left(X\right)+P\left(Y\right|\neg\:X\left)\:\right(1-P\left(X\right)]$$.

This approach allows a quantitative estimation of how the post-test probability of disease X varies as a function of both the prior probability of X and the conditional probability of observing Y among affected subjects, while accounting for the frequency of Y in the non-affected population. Final probabilities were reported as percentages and summarized in tables to allow direct comparison across the different scenarios analyzed.

### Sensitivity analysis

To evaluate the robustness of the probabilistic framework, a one-way sensitivity analysis was performed by varying the assumed pre-test probability of IGHD across a clinically plausible range (15%–45%), encompassing the prevalence values reported in the selected studies and accounting for potential differences in patient selection and stimulation testing methodology.

For each assumed pre-test probability, post-test probability was recalculated for all examined IGF-I SDS thresholds (≥ 0, ≥ 0.5, and ≥ 1) using the same conditional probabilities derived from the published IGF-I distributions. Analyses were conducted separately for AO-GHD and CO-GHD scenarios.

## Results

### Probability of IGHD at GH stimulation testing in the absence of additional pituitary hormone deficiencies according to literature data

In the study by Hartman et al. [17], 817 adults with a history of hypothalamic–pituitary disease were evaluated; 139 patients had no additional anterior pituitary hormone deficiencies apart from suspected GHD. Adult GHD was defined as a peak GH < 2.5 µg/L following GH stimulation testing, including arginine, L-Dopa, L-Dopa/propranolol, ITT, arginine/L-Dopa, L-Dopa/clonidine, insulin/GHRH tests. Among patients without other pituitary hormone deficiencies, the probability of an impaired GH response was 41%.

In the study by Toogood et al. [22], 190 adults with hypothalamic–pituitary disease were assessed, of whom 54 had no additional anterior pituitary hormone deficiencies. Adult GHD was defined as a peak GH < 2.5 µg/L during an ITT. In this subgroup, the probability of an impaired GH response was 24%.

In the study by Sassolas et al. [23], 549 adults with hypothalamic–pituitary disease were included; 190 patients had no additional anterior pituitary hormone deficiencies. Adult GHD was defined as a peak GH < 3 µg/L following GH stimulation testing, with the ITT performed in 75% of cases. In this subgroup, the probability of an impaired GH response was 20%.

### Distribution of IGF-I SDS values in patients with IGHD

Mean ± SD values of IGF-I SDS in patients with IGHD were derived from the KIMS database, specifically from the studies by Abs et al. [26] (Table 1) and Klose et al. [27] (Table 2).

**Table 1:** IGF-I SDS distribution and estimated percentiles in adult- and childhood-onset isolated GH deficiency (data derived from reference 26)

	AO-IGHD (*n* = 167)	CO-IGHD (*n* = 107)
IGF-I SDS (mean ± SD)	-1.4 ± 1.5	-3.4 ± 2.2
Percentile IGF-I SDS = 0	82°	94°
Percentile IGF-I SDS = 0.5	90°	96°
Percentile IGF-I SDS = 1	94.5°	97.7°

*AO-IGHD* adult-onset isolated GH deficiency, *CO-IGHD* childhood-onset isolated GH deficiency, *IGF-I* insulin-like growth factor I, *SD* standard deviation, *SDS* standard deviation score

**Table 2:** IGF-I SDS distribution and estimated percentiles in adult- and childhood-onset isolated GH deficiency (data derived from reference 27)

	AO-IGHD (*n* = 283)
IGF-I SDS (mean ± SD)	-1.3 ± 1.2
Percentile IGF-I SDS = 0	86°
Percentile IGF-I SDS = 0.5	93°
Percentile IGF-I SDS = 1	97°

*AO-IGHD* adult-onset isolated GH deficiency, *IGF-I* insulin-like growth factor I, *SD* standard deviation, *SDS* standard deviation score

The study by Abs et al. included 274 patients with IGHD, of whom 167 had AO-IGHD and 107 had CO-IGHD [26]. In the 167 patients with AO-IGHD, mean ± SD IGF-I SDS values were − 1.4 ± 1.5. IGF-I SDS values of 0, 0.5, and 1 corresponded to the 82nd, 90th, and 94.5th percentiles, respectively (Table 1). Accordingly, the probability of an IGF-I SDS ≥ 0, ≥ 0.5, or ≥ 1 in patients with AO-IGHD was estimated to be 18%, 10%, and 5.5%, respectively.

In the 107 patients with CO-IGHD, mean ± SD IGF-I SDS values were − 3.4 ± 2.2. IGF-I SDS values of 0, 0.5, and 1 corresponded to the 94th, 96th, and 97.7th percentiles, respectively (Table 1). Based on these data, the probability of an IGF-I SDS ≥ 0, ≥ 0.5, or ≥ 1 in patients with CO-IGHD was estimated to be 6%, 4%, and 2.3%, respectively.

The study by Klose et al. included 283 patients with AO-IGHD [27]. Mean ± SD IGF-I SDS values were − 1.3 ± 1.2. IGF-I SDS values of 0, 0.5, and 1 corresponded to the 86th, 93rd, and 97th percentiles, respectively (Table 2). Accordingly, the probability of an IGF-I SDS ≥ 0, ≥ 0.5, or ≥ 1 in this cohort was estimated to be 14%, 7%, and 3%, respectively.

### Estimated probability of IGHD in the absence of additional pituitary hormone deficiencies and in the presence of IGF-I SDS ≥ 0, ≥ 0.5, or ≥ 1

Table 3 summarizes the estimated probabilities of IGHD in patients without additional pituitary hormone deficiencies and with IGF-I SDS values ≥ 0, ≥ 0.5, or ≥ 1.

**Table 3:** Estimated probability of isolated GHD by IGF-I SDS Cut-offs in patients without additional pituitary deficiencies

	Probability of IGHD in the absence of other pituitary deficiencies		
Probability of IGF-I SDS ≥ 0 in AO-IGHD and CO-IGHD	41% [17]	24% [22]	20% [23]
AO-IGHD 18% [26]	19.8%	10.2%	8.3%
AO-IGHD 14% [27]	16.3%	8.1%	6.5%
CO-IGHD 6% [26]	7.7%	3.6%	2.9%
Probability of IGF-I SDS ≥ 0.5 in AO-IGHD and CO-IGHD			
AO-IGHD 10% [26]	18.3%	9.2%	7.5%
AO-IGHD 7% [27]	13.6%	6.7%	5.3%
CO-IGHD 4% [26]	8.2%	3.9%	3.1%
Probability of IGF-I SDS ≥ 1 in AO-IGHD and CO-IGHD			
AO-IGHD 5.5% [26]	19.3%	9.8%	7.9%
AO-IGHD 3% [27]	11.5%	5.6%	4.5%
CO-IGHD 2.3% [26]	9.1%	4.3%	3.5%

*AO-IGHD* adult-onset isolated GH deficiency, *CO-IGHD* childhood-onset isolated GH deficiency, *IGF-I* insulin-like growth factor I, *IGHD* isolated GH deficiency, *SDS* standard deviation score

In patients with hypothalamic–pituitary disease, no additional pituitary hormone deficiencies, and IGF-I SDS ≥ 0, the estimated probability of IGHD ranged from a maximum of 7.7% to a minimum of 2.9% in adults with CO-IGHD, and from a maximum of 19.8% to a minimum of 6.5% in adults with AO-IGHD.

When considering an IGF-I SDS cut-off ≥ 0.5, the estimated probability of IGHD ranged from 8.2% to 3.1% in adults with CO-IGHD and from 18.3% to 5.3% in adults with AO-IGHD.

Finally, in the presence of IGF-I SDS ≥ 1, the estimated probability of IGHD ranged from 9.1% to 3.5% in adults with CO-IGHD and from 19.3% to 4.5% in adults with AO-IGHD.

### Sensitivity analysis

When varying the assumed pre-test probability between 15% and 45%, post-test probability in patients with IGF-I SDS ≥ 0 remained consistently reduced in both AO-GHD and CO-GHD models.

In the AO-GHD scenario, post-test probability ranged approximately from 6% to 23%, while in the CO-GHD model it remained even lower across the same prevalence range. Notably, when baseline prevalence was ≤ 30%, post-test probability remained close to or below 15% in both models.

These findings indicate that the probability-lowering effect associated with IGF-I SDS ≥ 0 is maintained across a broad range of clinically plausible baseline risk assumptions.

Higher IGF-I SDS thresholds (≥ 0.5 and ≥ 1) were associated with further reductions in post-test probability across all examined prevalence scenarios (data not shown), consistent with the monotonic behavior of the model.

## Discussion

In this study, we used a Bayesian mathematical model to estimate the probability of GHD in adults with hypothalamic–pituitary disease but no additional pituitary hormone deficiencies, based on IGF-I SDS levels. These findings provide a framework for a more rational diagnostic approach in this clinical setting, with the potential to reduce unnecessary dynamic testing. From a methodological perspective, this model should be interpreted as a clinical decision-support framework rather than a direct description of empirical distributions. Specifically, based on literature data on the probability of an impaired response to GH stimulation testing in patients with a history of hypothalamic–pituitary disease and no additional pituitary hormone deficiencies, we demonstrated that: (i) the presence of IGF-I SDS levels ≥ 0 is associated with a risk of IGHD ranging from 6.5% to 19.8% in cases of AO-IGHD and from 2.9% to 7.7% in cases of CO-IGHD; (ii) IGF-I SDS levels ≥ 0.5 are associated with a risk of IGHD ranging from 5.3% to 18.3% in AO-IGHD and from 3.1% to 8.2% in CO-IGHD; and (iii) IGF-I SDS levels ≥ 1 are associated with a risk of IGHD ranging from 4.5% to 19.3% in AO-IGHD and from 3.5% to 9.1% in CO-IGHD. The present study represents, to our knowledge, the first attempt to formally develop a probabilistic model aimed at excluding, rather than confirming, the diagnosis of adult GHD without the use of GH stimulation tests. While current diagnostic strategies already allow selected patients to be diagnosed with GHD without dynamic testing—namely those presenting with multiple additional pituitary hormone deficiencies, with or without markedly reduced IGF-I levels [4–7]—no previous approach has specifically addressed the opposite clinical scenario: identifying a subgroup of patients in whom the likelihood of GHD is sufficiently low to reasonably avoid stimulation testing. This distinction is clinically relevant. Existing guidelines endorse a “rule-in” strategy for GHD, whereby the presence of at least three additional pituitary hormone deficiencies confers a very high pre-test probability of GHD, estimated to range between approximately 90% and 96%, thus justifying the diagnosis without further testing [4–7, 17, 22, 23]. In contrast, patients with hypothalamic–pituitary disease but preserved anterior pituitary function represent a diagnostically challenging group, in whom dynamic testing is routinely recommended despite the well-recognized limitations of currently available GH stimulation tests [2, 3, 13–15]. The present work proposes a complementary and conceptually novel “rule-out” approach, designed to simplify the diagnostic pathway in this specific clinical context.

These considerations may be particularly relevant in adult GHD. Pituitary disorders are more prevalent from the fifth decade onwards, and in this population the indication for GH replacement therapy requires careful evaluation of the risk–benefit balance. In this context, a probabilistic approach integrating IGF-I SDS with clinical pre-test probability may support more individualized and cautious decision-making.

Using a Bayesian framework, we integrated literature-derived estimates of (i) the probability of an impaired GH response to stimulation tests in patients with hypothalamic–pituitary disease and no additional pituitary hormone deficiencies, and (ii) the probability that patients with IGHD present IGF-I SDS values above predefined thresholds. This approach allowed estimation of the post-test probability of GHD in patients characterized by the simultaneous absence of other pituitary deficits and relatively preserved IGF-I levels.

Across all proposed models, the estimated probability of GHD consistently remained below 20%, regardless of the IGF-I SDS cut-off considered (≥ 0, ≥ 0.5, or ≥ 1), and irrespective of whether GHD was of childhood or adult onset. This consistency across different assumptions and scenarios represents a relevant finding of the study. Importantly, these probability estimates must be interpreted in the context of clinical decision-making rather than as absolute diagnostic thresholds.

In adult GHD, a certain degree of diagnostic uncertainty is unavoidable [2]. GH stimulation tests themselves are affected by limited reproducibility, assay variability, and imperfect sensitivity and specificity, with results influenced by age, body mass index, and testing methodology [2, 3, 13–15]. This is further supported by the marked variability in the prevalence of pathological responses reported by Hartman et al. (41%) [17], where the ITT was applied in only a minority of cases, compared with the studies by Toogood [22] and Sassolas [23], in which the ITT was used as the sole stimulation test in the former and in the majority of patients (75%) in the latter, suggesting a relevant impact of test selection on diagnostic outcomes [17, 22, 23].

Moreover, it should be acknowledged that the clinical consequences of diagnostic error are asymmetric. While failure to diagnose true GHD may delay the initiation of replacement therapy, incorrectly attributing GHD to an otherwise healthy individual exposes the patient to long-term recombinant human GH treatment, which is costly, requires lifelong daily injections, and may be associated with adverse effects. In this context, a residual probability of GHD below 20% may reasonably be considered acceptable to support a decision not to pursue further diagnostic testing. From a healthcare system perspective, reducing unnecessary GH stimulation tests may also have important implications, as these procedures are resource-intensive, time-consuming, and not without risks or contraindications. A more selective use of dynamic testing based on post-test probability estimates could therefore improve cost-effectiveness while reducing patient burden.

Moreover, it should be emphasized that when the proposed model is based on the probability of an impaired response to GH stimulation testing derived predominantly [23] or exclusively [22] from the use of the ITT—currently regarded as the gold standard for the diagnosis of GHD [7, 14]—the residual probability of GHD is substantially below 10%, regardless of the IGF-I SDS level considered.

In this specific clinical context—namely adults with hypothalamic–pituitary disease, preserved anterior pituitary function, and IGF-I SDS values within the normal range—GH stimulation testing may provide limited additional diagnostic information. When post-test probability remains consistently low after integration of clinical and biochemical parameters, the incremental value of dynamic testing in guiding clinical decision-making may be marginal.

Among the three models evaluated, the approach based on an IGF-I SDS cut-off ≥ 0 emerges as the most clinically reasonable and pragmatic. Although higher cut-offs (≥ 0.5 or ≥ 1) further reduce the estimated probability of GHD, the absolute differences in post-test probability are modest. In contrast, an IGF-I SDS ≥ 0 identifies a substantially larger proportion of patients who could potentially avoid stimulation testing, thereby maximizing the primary objective of this study: simplification of the diagnostic pathway. From a practical standpoint, this threshold reflects relatively preserved GH–IGF-I axis function and identifies individuals with relatively preserved IGF-I levels within the upper part of the normal distribution, in whom the likelihood of clinically relevant GHD appears particularly low.

**Fig. 2:**
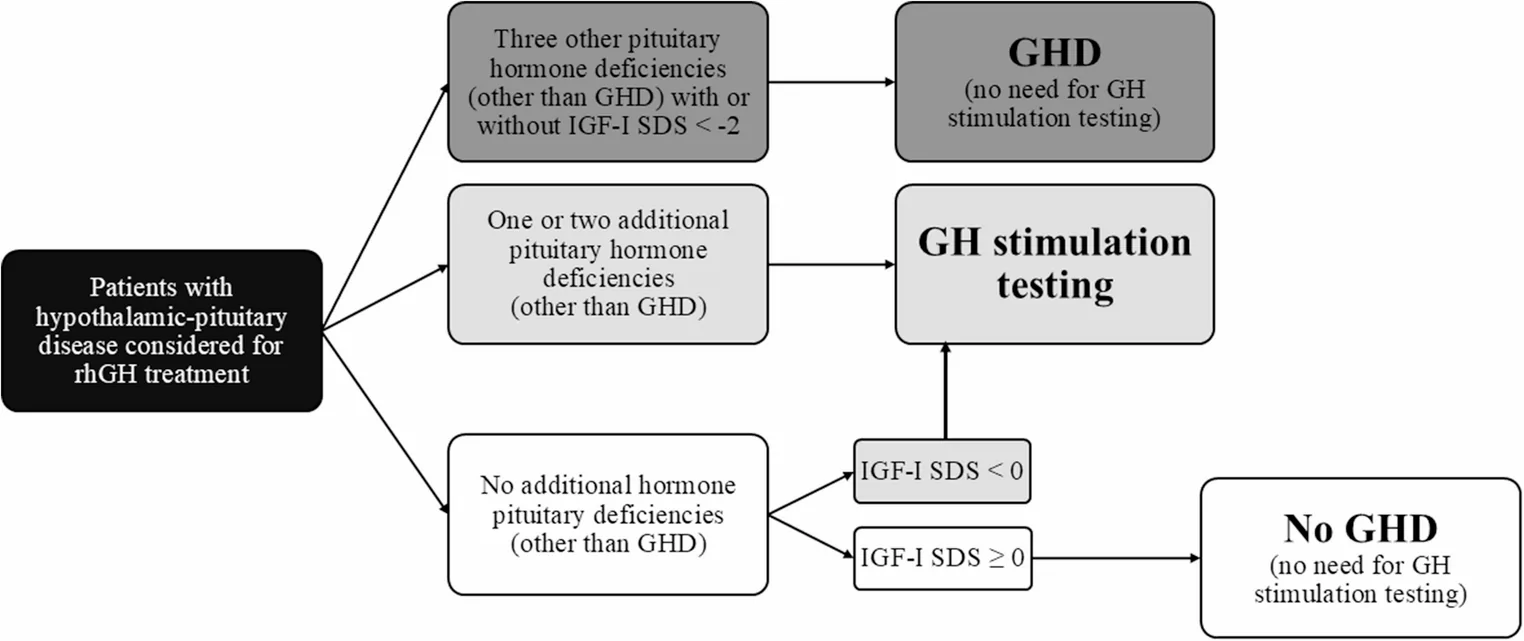
Proposed diagnostic flowchart for adult GHD based on pituitary function, IGF-I SDS, and GH stimulation testing

On the basis of these findings and considerations, we propose a new diagnostic flowchart for adult GHD (Fig. 2). Consistent with current guidelines, in patients with hypothalamic–pituitary disease who present with three additional pituitary hormone deficiencies, apart from the suspected GHD, the use of dynamic diagnostic testing appears unnecessary, as the presence of GHD can be assumed [4–7]. Similarly, as demonstrated by the present study, it is reasonable to exclude the diagnosis of GHD without performing stimulation tests in patients with a history of hypothalamic–pituitary disease, in the absence of other pituitary hormone deficiencies apart from the suspected GHD, and with IGF-I SDS levels ≥ 0. In contrast, the need for stimulation testing for the diagnosis of GHD remains valid in all other patients, namely those with one or two additional pituitary hormone deficiencies apart from the suspected GHD, regardless of IGF-I SDS levels, as well as those without other pituitary hormone deficiencies apart from the suspected GHD but with IGF-I SDS levels < 0.

The stability of the model across varying baseline risk assumptions further supports its conceptual validity. Sensitivity analysis demonstrated that the probability-reducing effect of IGF-I SDS ≥ 0 persisted across a wide range of pre-test probability values in both AO-GHD and CO-GHD scenarios.

These findings suggest that the model is robust to moderate variations in baseline assumptions and, indirectly, to potential deviations from idealized distributional conditions. Even under relatively high prevalence assumptions, post-test probability remained substantially lower than baseline risk, and approached or fell below clinically relevant thresholds when baseline prevalence was moderate.

Although higher IGF-I SDS thresholds (≥ 0.5 and ≥ 1) were associated with even lower post-test probabilities, the primary clinical implication of the model is already evident at the ≥ 0 threshold, supporting the potential role of a probability-based refinement of patient selection for dynamic testing.

This study has several limitations that should be acknowledged. First, the analysis is based on aggregated data derived from previously published studies rather than individual patient-level datasets. Consequently, it was not possible to directly assess the empirical distribution of IGF-I SDS in GHD and non-GHD populations or to formally test distributional assumptions, such as normality, using statistical methods (e.g., Shapiro–Wilk test). It should also be emphasized that IGF-I SDS values are standardized with respect to a healthy reference population and are therefore expected to follow a normal distribution only in that context. In contrast, the present analysis focuses on the distribution of IGF-I SDS within populations of patients with IGHD, in whom the distribution is characteristically shifted toward lower values and may differ in dispersion. Accordingly, the estimation of percentiles corresponding to predefined IGF-I SDS thresholds (≥ 0, ≥ 0.5, ≥ 1) was based on the distributional parameters reported in GHD cohorts, rather than on the theoretical properties of SDS in the reference population. This distinction is essential to correctly interpret the probabilistic framework adopted in this study. Although deviations from normality cannot be excluded, moderate departures from normality are unlikely to substantially affect the estimated tail probabilities, particularly given the exploratory and probabilistic nature of the present model. Importantly, the aim of the present analysis was not to provide precise percentile estimates, but rather to obtain clinically meaningful approximations to inform a Bayesian probability framework.

Second, the proposed model has not been prospectively validated in an independent cohort. Therefore, the findings should be considered hypothesis-generating and require confirmation in real-world clinical settings. Prospective studies based on individual patient-level data are needed both to validate the underlying assumptions and to refine the probabilistic estimates, as well as to assess the performance, safety, and clinical impact of the proposed rule-out strategy before its routine clinical implementation.

A major strength of this study lies in the use of data derived from independent and well-established sources. Estimates of pre-test probability were obtained from previously published studies conducted in different cohorts and independent research settings, none of which are related to the present work, thereby reducing the risk of methodological circularity. In addition, the distribution of IGF-I SDS values in patients with IGHD was derived from the KIMS database, one of the largest post-marketing surveillance registries in adult GHD. The integration of these independent datasets enhances the robustness and generalizability of the proposed probabilistic model.

In conclusion, this study introduces a proof-of-concept probabilistic framework for ruling out adult GHD without stimulation testing in a well-defined clinical setting. By identifying patients with a low likelihood of disease, this approach may have the potential to simplify diagnostic workflows, reduce patient burden, and optimize healthcare resource utilization, while maintaining an acceptable level of diagnostic safety.

Future studies based on individual patient data are needed to validate these assumptions and to further refine the proposed probabilistic model.

## Data Availability

All data analyzed in this study were extracted from previously published articles, which are cited in the reference list. The dataset constructed for the purpose of the present analysis is available from the corresponding author upon reasonable request.
